# Dipotassium dialuminium cyclo­octa­phosphate

**DOI:** 10.1107/S1600536810020751

**Published:** 2010-06-05

**Authors:** Abdelghani Oudahmane, Daniel Avignant, Daniel Zambon

**Affiliations:** a Université Cadi Ayyad, Laboratoire de la Matière Condensée et de l’Environnement, Faculté des Sciences Semlalia, Département de Chimie, BP 2390, 40000, Marrakech, Morocco; b Université Blaise Pascal, Laboratoire des Matériaux Inorganiques, UMR CNRS 6002, 24 Avenue des Landais, 63177 Aubière, France

## Abstract

Single crystals of the title compound, K_2_Al_2_P_8_O_24_, were obtained by solid-state reaction. The monoclinic structure is isotypic with that of the Ga^III^ analogue and is built of eight-membered phosphate ring anions P_8_O_24_^8−^ (2/*m* symmetry) isolated from each other and further linked by isolated AlO_6_ octa­hedra (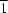 symmetry) by sharing corners. Each AlO_6_ octa­hedron is linked to four P_8_O_24_^8−^ rings in such a way that two rings are linked through bidentate diphosphate groups attached in the *cis* positions on two opposite parallel edges of the octa­hedron. The two other rings are linked *via* corner-sharing to the two remaining corners in the *trans* positions of the AlO_6_ octa­hedron. Each P_8_O_24_^8−^ ring anion is linked to eight AlO_6_ octa­hedra. More accurately, each ring anion is linked to four AlO_6_ octa­hedra through bidentate diphosphate groups attached in the *cis* positions to the AlO_6_ octa­hedron and to the four remaining octa­hedra by sharing corners. This three-dimensional linkage delimits channels running parallel to [001] in which the ten-coordinated K^+^ cations (2 symmetry) are distributed over two columns. These columns alternate with empty octa­gonally-shaped channels expanding through the P_8_O_24_^8−^ ring anions.

## Related literature

The synthesis and an approximate unit cell with a slightly smaller *β* angle were reported for the title compound more than a quarter of a century ago (Grunze *et al.*, 1983 ▶). The crystal structures of isotypic compounds determined from single-crystal data have been reported for K_2_Ga_2_P_8_O_24_ (Palkina *et al.*, 1979 ▶) and K_2_Mn_2_P_8_O_24_ (Murashova & Chudinova, 1999 ▶). The isostructural potassium-containing cyclo­octa­phosphates K_2_V_2_P_8_O_24_ (Lavrov *et al.*, 1981 ▶), K_2_Fe_2_P_8_O_24_ (Grunze *et al.*, 1983 ▶) and K_2_Cr_2_P_8_O_24_ (Grunze & Chudinova, 1988 ▶) were reported without detailed structure analyses. For a review of the crystal chemistry of cyclo­octa­phosphates, see: Durif (1995 ▶, 2005 ▶). For potential applications of aluminophosphates, see: Cheetham *et al.* (1999 ▶); Hartmann & Kevan (1999 ▶). For background to distortion indices, see: Momma & Izumi (2008 ▶).

## Experimental

### 

#### Crystal data

K_2_Al_2_P_8_O_24_
*M* *_r_* = 763.92
Monoclinic, 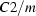

*a* = 16.598 (2) Å
*b* = 12.2150 (17) Å
*c* = 5.0705 (7) Å
β = 100.315 (4)°
*V* = 1011.4 (2) Å^3^
*Z* = 2
Mo *K*α radiation
μ = 1.31 mm^−1^
*T* = 296 K
0.30 × 0.10 × 0.08 mm

#### Data collection

Bruker APEXII CCD diffractometer
Absorption correction: multi-scan (*SADABS*; Bruker, 2008 ▶) *T*_min_ = 0.562, *T*_max_ = 0.748
9643 measured reflections
2844 independent reflections
2252 reflections with *I* > 2σ(*I*)
*R*_int_ = 0.047

#### Refinement

*R*[*F* ^2^ > 2σ(*F* ^2^)] = 0.042
*wR*(*F* ^2^) = 0.124
*S* = 1.07
2844 reflections
87 parameters
Δρ_max_ = 1.40 e Å^−3^
Δρ_min_ = −1.04 e Å^−3^

### 

Data collection: *APEX2* (Bruker, 2008 ▶); cell refinement: *SAINT* (Bruker, 2008 ▶); data reduction: *SAINT*; program(s) used to solve structure: *SHELXS97* (Sheldrick, 2008 ▶); program(s) used to refine structure: *SHELXL97* (Sheldrick, 2008 ▶); molecular graphics: *DIAMOND* (Brandenburg, 1999 ▶), CaRine (Boudias & Monceau, 1998 ▶) and *ORTEP-3* (Farrugia, 1997 ▶); software used to prepare material for publication: *SHELXTL* (Sheldrick, 2008 ▶).

## Supplementary Material

Crystal structure: contains datablocks I, global. DOI: 10.1107/S1600536810020751/wm2355sup1.cif

Structure factors: contains datablocks I. DOI: 10.1107/S1600536810020751/wm2355Isup2.hkl

Additional supplementary materials:  crystallographic information; 3D view; checkCIF report

## Figures and Tables

**Table 1:** Selected bond lengths (Å)

P1—O1^i^	1.4861 (12)
P1—O3	1.4956 (12)
P1—O4	1.5701 (13)
P1—O7	1.5794 (8)
P2—O5	1.4622 (14)
P2—O2	1.4983 (12)
P2—O6	1.5957 (10)
P2—O4	1.6070 (13)

Symmetry code: (i) 
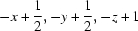
.

**Fig. 1.**
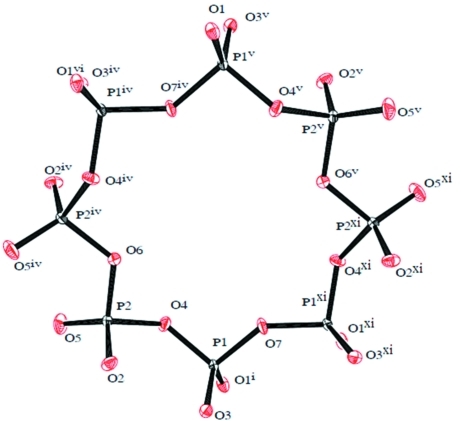
ORTEP-3 view (Farrugia, 1997) of the centrosymmetric (P8O24)8- ring anion. Displacement ellipsoids are drawn at the 50% probability level. Symmetry codes: (i) -x, -y + 1, -z + 1; (iv) -x, y, -z; (v) -x, -y + 1, -z; (vi) x - 1/2, -y + 1/2, z; (ix) -x + 1/2, -y + 1/2, -z + 1; (xi) x, -y, z.

**Fig. 2.**
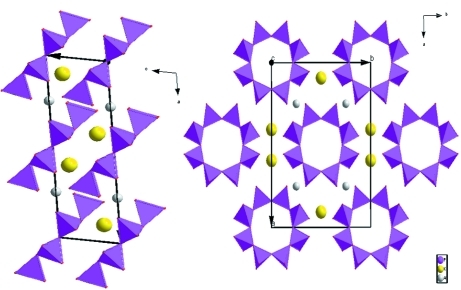
Projections of the 8-membered ring anions P8O248- along [010] (left) and [001] (right).

**Fig. 3.**
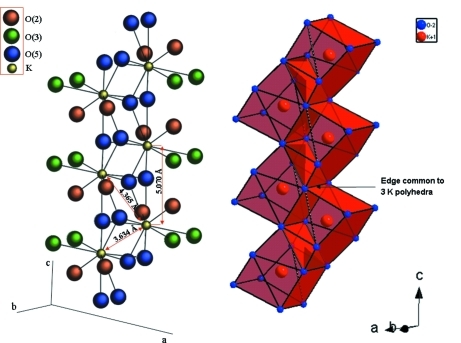
Partial view showing the packing of the K—O polyhedra along the c axis in the ball and stick (left) and polyhedral (right) representation.

**Fig. 4.**
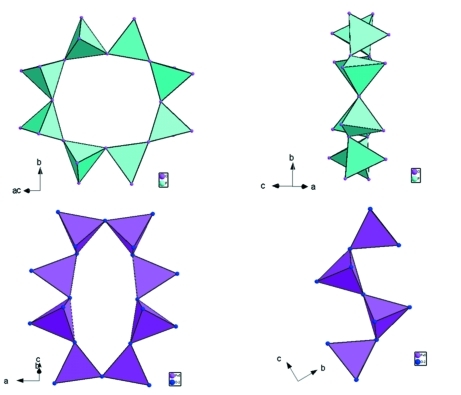
Comparison of the 8-membered ring anions P8O248- (both with internal 2/m symmetry) in K2Al2P8O24 (top) and Ag9NaP8O24(NO3)2.4H2O (bottom).

## supplementary crystallographic information

### Comment

The title compound, K_2_Al_2_P_8_O_24_, belongs to porous crystalline open-framework materials that gain growing interest for their potential applications as molecular sieves or catalysts (Cheetham *et al.* 1999; Hartmann & Kevan, 1999). The title cyclooctaphosphate is isotypic with K_2_Ga_2_P_8_O_24_ (Palkina *et al.*, 1979) and K_2_Mn_2_P_8_O_24_ (Murashova & Chudinova, 1999). This structural family also includes K_2_V_2_P_8_O_24_ (Lavrov *et al.*, 1981), K_2_Fe_2_P_8_O_24_ (Grunze *et al.*, 1983) and K_2_Cr_2_P_8_O_24_ (Grunze & Chudinova, 1988), the structures of which have not yet been refined from X-ray diffraction data.

The crystal structure of K_2_*M*_2_P_8_O_24_ is built up of 8-membered phosphate ring anions P_8_O_24_^8-^ (symmetry 2/*m*) (Fig. 1), further linked by isolated *M*^III^O_6_ octahedra to form the three-dimensional skeleton. Each *M*O_6_ octahedron is linked to four ring anions P_8_O_24_^8-^. Two ring anions are linked by corner-sharing in *trans* positions whereas the two others are connected in a bidentate fashion in *cis* positions on two opposite edges of the equatorial plane of the octahedron. This three-dimensional framework of K_2_Al_2_P_8_O_24_ delimits two kinds of channels expanding along the [001] direction (Fig. 2). The first channel is octagonally shaped since passing through the ring anions and is empty despite a size of ≈ 5.2 Å in diameter. The second channel, cross shaped, accommodates the K^+^ ions in a [6 + 4] coordination. The K^+^ ions are located over two columns shifted of about *c*/2 with respect to each other along the *c* axis. Thus they form two distinct K—K pairs with common square faces involving only O(5) oxygen atoms. In the first pair, corresponding to the shortest K—K distance (3.634 (9) Å) (Fig. 3), the four O(5) atoms delimit a pseudo-square face with O—O separations of 2.995 (2) and 3.064 (6) Å and O(5)—O(5)—O(5) angles of 90°. This pair corresponds to the two shortest K—O distances, *viz.* 2.7559 (15) and 2.8612 (15) Å. In the second pair with a K—K distance of 4.365 (7) Å, the four O(5) atoms at the vertices of the pseudo-square face (O—O separation 2.995 (2) and 3.139 (7) Å with O(5)—O(5)—O(5) angles of 90°), the respective K—O bonds lengths are 2.8612 (15) and 3.2790 (17) Å. The polyhedra surrounding three potassium cations engaged in two successive pairs (one long and one short) form a cluster within they share a common O(5)—O(5) edge with a contact distance of 2.995 (2) Å. This potassium-oxygen polyhedra packing also prevails in the Ga and Mn cyclooctaphosphate analogues but the K—O distances spread over larger ranges, *viz.* from 2.754 (28) Å to 3.359 (28) Å and from 2.738 (2) to 3.506 (2) Å, respectively. Thus the respective coordinations of the potassium cations can be regarded as being [6 + 4] and [8 + 2] for Ga and Mn cyclooctaphosphate.

A careful examination of the geometry of the *M*^III^O_6_ octahedra in this structural type shows that the distortion index (bond length) (Momma & Izumi, 2008) increases from Al to Mn (0.0117 for Al, 0.0202 for Ga and 0.0574 for Mn). The AlO_6_ octahedron is only very slightly distorted with two shorter Al—O distances of 1.8523 (12) Å and four others very close to 1.90 Å (1.9013 (12) (× 2) and 1.9021 (11) (× 2) Å). The significant larger distortion of the MnO_6_ octahedron is probably due to the Jahn-Teller effect associated with the *d*^4^ electronic configuration of Mn^III^ (Murashova & Chudinova, 1999).

Besides the structural family to which the title compound belongs, only another sodium and silver- containing cyclooctaphosphate, Ag_9_NaP_8_O_24_(NO_3_)_2_.4H_2_O, exhibits a ring anion with internal 2/*m* symmetry among the presently known cyclooctaphosphates (Durif, 1995, 2005). However, despite the common internal symmetry, the shape of the 8-membered ring anion present in this structure is very different from that of the title compound as shown in Fig. 4.

### Experimental

Single-crystals of the title compound were obtained by solid state reaction, from the reagents K_2_CO_3_, Al_2_O_3_ and (NH_4_)H_2_PO_4_ in the molar ratio K / P / Al = 57 / 34 / 9. The mixture has progressively been heated up to 873 K over a period of 12 h. Then the temperature was slowly decreased down to 723 K at the rate of 5 K h^-1^ and maintained at this value for 12 h. Then a new cooling step down to 573 K at the rate of 5 K h^-1^ was carried out before the furnace was switched off. Single-crystals of K_2_Al_2_P_8_O_24_ were extracted from the batch by washing with hot water in order to remove the excess of P_2_O_5_. A translucent colorless needle of the title compound was used for the structure refinement.

### Refinement

The highest residual peak in the final difference Fourier map was located 0.74 Å from atom P2 and the deepest hole was located 0.69 Å from atom P1.

### Crystal data

**Table:**

K_2_Al_2_P_8_O_24_	*F*(000) = 752
*M**_r_* = 763.92	*D*_x_ = 2.508 Mg m^−^^3^
Monoclinic, *C*2/*m*	Mo *K*α radiation, λ = 0.71073 Å
Hall symbol: -C 2y	Cell parameters from 3017 reflections
*a* = 16.598 (2) Å	θ = 3.3–37.7°
*b* = 12.2150 (17) Å	µ = 1.31 mm^−^^1^
*c* = 5.0705 (7) Å	*T* = 296 K
β = 100.315 (4)°	Needle, colourless
*V* = 1011.4 (2) Å^3^	0.30 × 0.10 × 0.08 mm
*Z* = 2	

### Data collection

**Table:**

Bruker APEXII CCD diffractometer	2844 independent reflections
Radiation source: fine-focus sealed tube	2252 reflections with *I* > 2σ(*I*)
graphite	*R*_int_ = 0.047
Detector resolution: 8.3333 pixels mm^-1^	θ_max_ = 38.7°, θ_min_ = 4.1°
ω and φ scans	*h* = −29→28
Absorption correction: multi-scan (*SADABS*; Bruker, 2008)	*k* = −18→20
*T*_min_ = 0.562, *T*_max_ = 0.748	*l* = −8→8
9643 measured reflections	

### Refinement

**Table:**

Refinement on *F*^2^	0 restraints
Least-squares matrix: full	Primary atom site location: structure-invariant direct methods
*R*[*F*^2^ > 2σ(*F*^2^)] = 0.042	Secondary atom site location: difference Fourier map
*wR*(*F*^2^) = 0.124	*w* = 1/[σ^2^(*F*_o_^2^) + (0.0738*P*)^2^ + 0.0398*P*] where *P* = (*F*_o_^2^ + 2*F*_c_^2^)/3
*S* = 1.07	(Δ/σ)_max_ < 0.001
2844 reflections	Δρ_max_ = 1.40 e Å^−^^3^
87 parameters	Δρ_min_ = −1.04 e Å^−^^3^

### Special details

**Table:**

Geometry. All e.s.d.'s (except the e.s.d. in the dihedral angle between two l.s. planes) are estimated using the full covariance matrix. The cell e.s.d.'s are taken into account individually in the estimation of e.s.d.'s in distances, angles and torsion angles; correlations between e.s.d.'s in cell parameters are only used when they are defined by crystal symmetry. An approximate (isotropic) treatment of cell e.s.d.'s is used for estimating e.s.d.'s involving l.s. planes.
Refinement. Refinement of *F*^2^ against ALL reflections. The weighted *R*-factor *wR* and goodness of fit *S* are based on *F*^2^, conventional *R*-factors *R* are based on *F*, with *F* set to zero for negative *F*^2^. The threshold expression of *F*^2^ > σ(*F*^2^) is used only for calculating *R*-factors(gt) *etc*. and is not relevant to the choice of reflections for refinement. *R*-factors based on *F*^2^ are statistically about twice as large as those based on *F*, and *R*- factors based on ALL data will be even larger.

### Fractional atomic coordinates and isotropic or equivalent isotropic displacement parameters (Å^2^)

**Table:**

	*x*	*y*	*z*	*U*_iso_*/*U*_eq_	
K	−0.09371 (4)	0.5000	0.25194 (13)	0.02290 (13)	
Al	0.2500	0.2500	0.0000	0.00771 (13)	
P1	0.19023 (2)	0.12098 (3)	0.46962 (8)	0.00720 (9)	
P2	0.07782 (2)	0.27631 (3)	0.16477 (8)	0.00835 (9)	
O1	0.28060 (8)	0.36965 (10)	0.2366 (2)	0.0118 (2)	
O2	0.14059 (7)	0.28394 (11)	−0.0132 (2)	0.0132 (2)	
O3	0.24968 (7)	0.15349 (10)	0.2942 (2)	0.0105 (2)	
O4	0.10711 (7)	0.18408 (11)	0.3873 (2)	0.0122 (2)	
O5	0.05469 (9)	0.37739 (12)	0.2866 (3)	0.0184 (3)	
O6	0.0000	0.21471 (15)	0.0000	0.0140 (3)	
O7	0.16028 (11)	0.0000	0.3993 (3)	0.0125 (3)	

### Atomic displacement parameters (Å^2^)

**Table:**

	*U*^11^	*U*^22^	*U*^33^	*U*^12^	*U*^13^	*U*^23^
K	0.0299 (3)	0.0147 (3)	0.0240 (3)	0.000	0.0046 (2)	0.000
Al	0.0085 (3)	0.0083 (3)	0.0064 (3)	0.0001 (2)	0.0016 (2)	0.0006 (2)
P1	0.00956 (16)	0.00518 (17)	0.00672 (15)	0.00032 (12)	0.00110 (11)	0.00033 (11)
P2	0.00850 (17)	0.00632 (18)	0.01023 (16)	0.00094 (12)	0.00164 (12)	0.00031 (12)
O1	0.0186 (5)	0.0084 (5)	0.0078 (4)	−0.0009 (4)	0.0006 (4)	−0.0006 (3)
O2	0.0095 (5)	0.0177 (6)	0.0130 (5)	0.0020 (4)	0.0033 (4)	0.0049 (4)
O3	0.0104 (5)	0.0111 (5)	0.0105 (5)	0.0011 (4)	0.0037 (3)	0.0033 (4)
O4	0.0108 (5)	0.0125 (6)	0.0138 (5)	0.0033 (4)	0.0034 (4)	0.0051 (4)
O5	0.0247 (6)	0.0105 (6)	0.0198 (6)	0.0053 (5)	0.0030 (5)	−0.0045 (5)
O6	0.0096 (7)	0.0092 (8)	0.0217 (8)	0.000	−0.0011 (6)	0.000
O7	0.0177 (7)	0.0051 (7)	0.0129 (7)	0.000	−0.0021 (5)	0.000

### Geometric parameters (Å, °)

**Table:**

K—O5^i^	2.7559 (15)	Al—O3	1.9021 (11)
K—O5^ii^	2.7559 (15)	P1—O1^ix^	1.4861 (12)
K—O5	2.8612 (15)	P1—O3	1.4956 (12)
K—O5^iii^	2.8612 (15)	P1—O4	1.5701 (13)
K—O2^iv^	2.9493 (14)	P1—O7	1.5794 (8)
K—O2^v^	2.9493 (13)	P2—O5	1.4622 (14)
K—O3^vi^	3.2431 (13)	P2—O2	1.4983 (12)
K—O3^vii^	3.2431 (13)	P2—O6	1.5957 (10)
K—O5^iv^	3.2790 (17)	P2—O4	1.6070 (13)
K—O5^v^	3.2790 (17)	P2—K^v^	3.4928 (7)
K—P2^iv^	3.4927 (7)	O1—P1^ix^	1.4861 (12)
K—P2^v^	3.4927 (7)	O2—K^v^	2.9494 (13)
Al—O2^viii^	1.8523 (12)	O3—K^x^	3.2431 (13)
Al—O2	1.8523 (12)	O5—K^i^	2.7559 (15)
Al—O1^viii^	1.9013 (12)	O5—K^v^	3.2790 (17)
Al—O1	1.9013 (12)	O6—P2^iv^	1.5957 (10)
Al—O3^viii^	1.9021 (11)	O7—P1^xi^	1.5793 (8)
			
O5^i^—K—O5^ii^	65.84 (6)	O5—K—P2^v^	106.81 (4)
O5^i^—K—O5	99.41 (4)	O5^iii^—K—P2^v^	58.01 (3)
O5^ii^—K—O5	66.08 (6)	O2^iv^—K—P2^v^	119.65 (3)
O5^i^—K—O5^iii^	66.08 (6)	O2^v^—K—P2^v^	25.13 (2)
O5^ii^—K—O5^iii^	99.41 (4)	O3^vi^—K—P2^v^	129.83 (3)
O5—K—O5^iii^	63.13 (6)	O3^vii^—K—P2^v^	74.69 (2)
O5^i^—K—O2^iv^	147.08 (5)	O5^iv^—K—P2^v^	78.81 (3)
O5^ii^—K—O2^iv^	82.35 (4)	O5^v^—K—P2^v^	24.68 (2)
O5—K—O2^iv^	73.61 (4)	P2^iv^—K—P2^v^	102.94 (2)
O5^iii^—K—O2^iv^	130.89 (4)	O2^viii^—Al—O2	180.0
O5^i^—K—O2^v^	82.35 (4)	O2^viii^—Al—O1^viii^	90.00 (6)
O5^ii^—K—O2^v^	147.08 (5)	O2—Al—O1^viii^	89.99 (6)
O5—K—O2^v^	130.89 (4)	O2^viii^—Al—O1	89.99 (6)
O5^iii^—K—O2^v^	73.61 (4)	O2—Al—O1	90.01 (6)
O2^iv^—K—O2^v^	126.97 (6)	O1^viii^—Al—O1	180.00 (5)
O5^i^—K—O3^vi^	109.11 (4)	O2^viii^—Al—O3^viii^	91.53 (5)
O5^ii^—K—O3^vi^	72.48 (4)	O2—Al—O3^viii^	88.47 (5)
O5—K—O3^vi^	112.63 (4)	O1^viii^—Al—O3^viii^	91.12 (5)
O5^iii^—K—O3^vi^	171.88 (4)	O1—Al—O3^viii^	88.88 (5)
O2^iv^—K—O3^vi^	49.77 (3)	O2^viii^—Al—O3	88.47 (5)
O2^v^—K—O3^vi^	112.88 (4)	O2—Al—O3	91.52 (5)
O5^i^—K—O3^vii^	72.48 (4)	O1^viii^—Al—O3	88.88 (5)
O5^ii^—K—O3^vii^	109.11 (4)	O1—Al—O3	91.12 (5)
O5—K—O3^vii^	171.88 (4)	O3^viii^—Al—O3	180.0
O5^iii^—K—O3^vii^	112.63 (4)	O1^ix^—P1—O3	116.38 (7)
O2^iv^—K—O3^vii^	112.88 (4)	O1^ix^—P1—O4	109.98 (7)
O2^v^—K—O3^vii^	49.77 (3)	O3—P1—O4	110.70 (7)
O3^vi^—K—O3^vii^	70.64 (5)	O1^ix^—P1—O7	109.34 (8)
O5^i^—K—O5^iv^	154.67 (6)	O3—P1—O7	109.20 (8)
O5^ii^—K—O5^iv^	114.04 (5)	O4—P1—O7	99.98 (8)
O5—K—O5^iv^	61.04 (5)	O5—P2—O2	117.73 (9)
O5^iii^—K—O5^iv^	89.66 (4)	O5—P2—O6	111.73 (8)
O2^iv^—K—O5^iv^	47.65 (3)	O2—P2—O6	107.39 (6)
O2^v^—K—O5^iv^	98.27 (4)	O5—P2—O4	111.39 (8)
O3^vi^—K—O5^iv^	94.08 (3)	O2—P2—O4	108.11 (7)
O3^vii^—K—O5^iv^	126.82 (4)	O6—P2—O4	98.73 (7)
O5^i^—K—O5^v^	114.04 (5)	O5—P2—K^v^	69.45 (6)
O5^ii^—K—O5^v^	154.67 (6)	O2—P2—K^v^	56.71 (5)
O5—K—O5^v^	89.66 (4)	O6—P2—K^v^	101.18 (5)
O5^iii^—K—O5^v^	61.04 (5)	O4—P2—K^v^	157.99 (5)
O2^iv^—K—O5^v^	98.27 (4)	P1^ix^—O1—Al	133.95 (8)
O2^v^—K—O5^v^	47.65 (3)	P2—O2—Al	138.36 (8)
O3^vi^—K—O5^v^	126.82 (4)	P2—O2—K^v^	98.17 (6)
O3^vii^—K—O5^v^	94.08 (3)	Al—O2—K^v^	113.58 (5)
O5^iv^—K—O5^v^	54.36 (5)	P1—O3—Al	136.54 (8)
O5^i^—K—P2^iv^	155.10 (4)	P1—O3—K^x^	120.51 (6)
O5^ii^—K—P2^iv^	93.25 (3)	Al—O3—K^x^	101.05 (4)
O5—K—P2^iv^	58.01 (3)	P1—O4—P2	132.20 (8)
O5^iii^—K—P2^iv^	106.81 (4)	P2—O5—K^i^	141.08 (8)
O2^iv^—K—P2^iv^	25.13 (2)	P2—O5—K	134.77 (8)
O2^v^—K—P2^iv^	119.65 (3)	K^i^—O5—K	80.59 (4)
O3^vi^—K—P2^iv^	74.69 (2)	P2—O5—K^v^	85.87 (6)
O3^vii^—K—P2^iv^	129.83 (3)	K^i^—O5—K^v^	114.04 (5)
O5^iv^—K—P2^iv^	24.68 (2)	K—O5—K^v^	90.34 (4)
O5^v^—K—P2^iv^	78.81 (3)	P2—O6—P2^iv^	123.73 (12)
O5^i^—K—P2^v^	93.25 (3)	P1^xi^—O7—P1	138.66 (12)
O5^ii^—K—P2^v^	155.10 (4)		

Symmetry codes: (i) −*x*, −*y*+1, −*z*+1; (ii) −*x*, *y*, −*z*+1; (iii) *x*, −*y*+1, *z*; (iv) −*x*, *y*, −*z*; (v) −*x*, −*y*+1, −*z*; (vi) *x*−1/2, −*y*+1/2, *z*; (vii) *x*−1/2, *y*+1/2, *z*; (viii) −*x*+1/2, −*y*+1/2, −*z*; (ix) −*x*+1/2, −*y*+1/2, −*z*+1; (x) *x*+1/2, *y*−1/2, *z*; (xi) *x*, −*y*, *z*.
